# Qualitative and Quantitative Characteristics of Soil Microbiome of Barents Sea Coast, Kola Peninsula

**DOI:** 10.3390/microorganisms9102126

**Published:** 2021-10-10

**Authors:** Maria Korneykova, Dmitry Nikitin, Vladimir Myazin

**Affiliations:** 1 Agrarian Technological Institute, Peoples’ Friendship University of Russia (RUDN University), 117198 Moscow, Russia; 2 Institute of North Industrial Ecology Problems—Subdivision of the Federal Research Centre, Kola Science Centre, Russian Academy of Science, 184209 Apatity, Russia; myazinv@mail.ru; 3 Department of Biology and Biochemistry of Soils, V.V. Dokuchaev Soil Science Institute, 119017 Moscow, Russia; dimnik90@mail.ru

**Keywords:** Kola Peninsula, prokaryotes, fungi, biomass, quantitative PCR

## Abstract

The soil microbiome of the Barents Sea coast of the Kola Peninsula is here characterized for the first time. The content of copies of ribosomal genes of archaea, bacteria, and fungi was determined by real-time PCR. Reserves and structure of biomass of soil microorganisms such as total biomass of fungi and prokaryotes, length and diameter of mycelium of fungi and actinomycetes, proportion of mycelium in biomass, number of spores and prokaryotic cells, proportion of small and large fungal propagules, and morphology of mycobiota spores were determined. The largest number of ribosomal gene copies was found for bacteria (from 6.47 × 10^9^ to 3.02 × 10^11^ per g soil). The number of copies of ribosomal genes of fungi and archaea varied within 10^7^–10^9^ copies of genes/g soil. The biomass of microorganisms (prokaryotes and fungi in total) varied from 0.023 to 0.840 mg/g soil. The share of mycobiota in the microbial biomass ranged from 90% to 97%. The number of prokaryotes was not large and varied from 1.87 × 10^8^ to 1.40 × 10^9^ cells/g of soil, while the biomass of fungi was very significant and varied from 0.021 to 0.715 mg/g of soil. The length of actinomycete mycelium was small—from 0.77 to 88.18 m/g of soil, as was the length of fungal hyphae—an order of magnitude higher (up to 504.22 m/g of soil). The proportion of fungal mycelium, an active component of fungal biomass, varied from 25% to 89%. Most (from 65% to 100%) of mycobiota propagules were represented by specimens of small sizes, 2–3 microns. Thus, it is shown that, despite the extreme position on the mainland land of Fennoscandia, local soils had a significant number of microorganisms, on which the productivity of ecosystems largely depends.

## 1. Introduction

The relevance of studying polar regions is constantly increasing due to the rapid and sensitive response of Arctic ecosystems to global climate change [1]. Extreme climatic conditions, a low level of energy and mass exchange, and short food chains cause the extreme vulnerability of the Arctic nature. Recently, the anthropogenic influence in the polar latitudes has increased sharply, which has led to an inevitable increase in the amount of technogenic pollution of soils and sea areas. A change in the chemical and physical properties of soils inevitably leads to a change in the number, composition, and functional activity of soil microbiota, which ensures the self-cleaning ability of soils and the normal functioning of the ecosystem.

Most of the Arctic territories are located in remote areas with partial or complete lack of infrastructure, which greatly complicates their study. At the same time, it is very important to understand the potential of the Arctic ecosystems for understanding its existence and recovery under conditions of an ever-increasing anthropogenic load, as well as for identifying microorganisms with a biotechnological potential.

One of these areas is the Kola Peninsula, which is the subarctic part of Fennoscandia and the Scandinavian Peninsula, as well as the extreme continental land of northwestern Russia, almost the entire territory of which is located beyond the Arctic Circle in natural zones of southern tundra, forest tundra, and northern taiga [2].

Long-term studies of the chemical and biological properties of the region’s soils were carried out in the subaerial biotopes [3,4,5], while the subaqueous biotopes remain overlooked, although they have a significant length along the coast of the Barents Sea and are constantly under pressure from economic activities (construction of factories in the coastal zone, shipping, transportation cargo, and oil products, etc.)

Soil microorganisms carry out biogeochemical cycles and quickly respond to environmental changes [6]. For this reason, microorganisms perform many ecological functions of the soil and can serve as effective highly sensitive indicators for detecting anthropogenic disturbances [7].

Most of the soil microbiological studies on the Kola Peninsula were carried out using the classical microbiology methods (plating methods, etc.); therefore, it is very important to confirm or refute the previously put forward hypotheses using modern methods of molecular genetic analysis (in particular, quantitative real-time PCR). Modern research methods also make it possible to study new groups of microorganisms, such as archaea, which have not previously been studied by classical methods. In addition, this group is of great interest to science in view of poor knowledge of it.

The research aimed to investigate the microbial communities (the structure of the biomass of microorganisms and the content of ribosomal genes of prokaryotes and fungi) of the subaqueous soils of the Barents Sea coast, to compare them with the subaerial soils of the region, and to identify the potential.

## 2. Objects and Methods

### 2.1. Research Area

In the northwest of the peninsula, the climate is arctic maritime; in other parts it is more continental and moderately cold. Summer is cool, winter, thanks to the influx of heat from the Barents Sea, is very warm for its latitude. The average annual temperature is about 0 °C, the average temperature of the warmest month (July) is from 8 to 12 °C, the coldest (February) is from −8 to −11 °C. Thaws occur throughout the winter. Frosts are possible for most of the growing season; snow can fall during any month. The frost-free period is about 110–120 days. The average annual rainfall is about 375 mm. Characteristic features of the climate are frequent strong winds and very high air humidity (up to 95–100%) [4,8].

Relief of the Kola Peninsula is represented by denudation-tectonic and denudation flat-topped mountain ranges, hills, low ridges, plateaus, and low basement plains with moraine ridges and lakes [8]. The highest mountain peaks (up to 1200 m) are noted in the western part of the peninsula, while the eastern part is much lower (150–250 m). In general, the Kola Peninsula is dominated by hilly elevations and plateaus. The geological basis of the country is formed by Archean and Proterozoic strata (Baltic Shield)—granites, gneisses, quartzites, crystalline schists, marble, and sandstones [9]. However, parent rocks of the Kola Peninsula are mainly represented by Quaternary deposits, among which moraine and fluvioglacial sandy and boulderer soils prevail [10].

On the coasts of the Murmansk region, within the tundra zone and forest–tundra, marching communities are found only fragmentarily since the geomorphological features of the coasts are unfavorable for the formation of halophytic vegetation. Communities of sandy and pebble beaches along the entire coast of the Murmansk region, both in the tundra and taiga zones, belong to the Honckenyo–Elymion arenariae group (Fernandez-Galiano 1954) Tx. 1966 (order Honckenyo–Elymetalia arenariae Tx. 1966, class Honckenyo–Elymetea arenariae Tx. 1966). The beach communities of the Murmansk coast are typical of the subarctic coasts in the tundra and forest–tundra subzones and belong to syntaxa of predominantly subarctic, boreal, and moderate distribution, which gives the considered areas commonality with more southerly boreal territories [11]. 

### 2.2. Site Description

**Figure 1:**
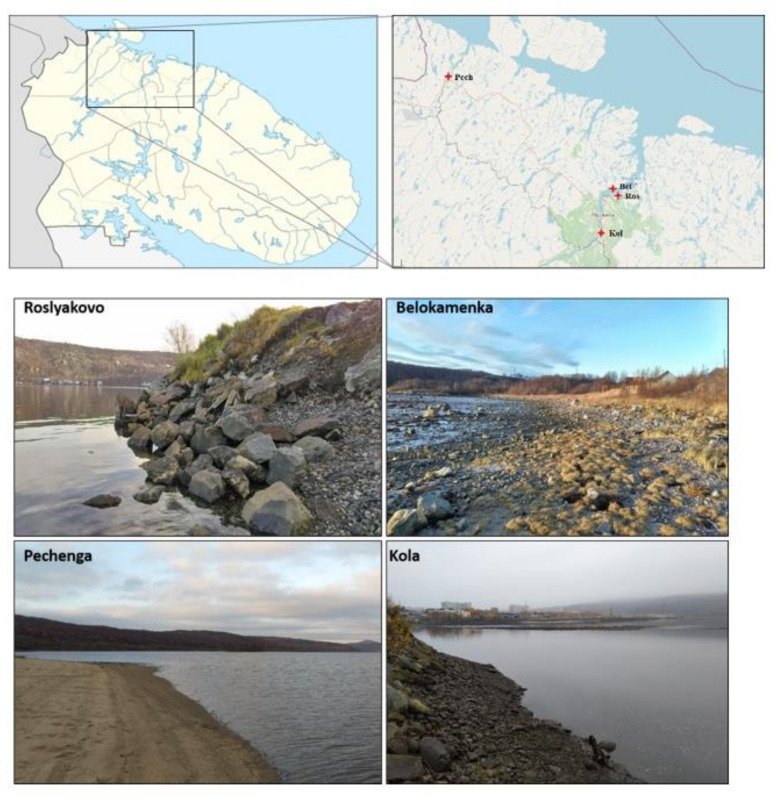
Sampling points.

To avoid inaccuracies and confusion, in this article we distinguish between “object” and “sample”. Objects are selected territories. Samples are small amounts of soil taken from the objects of study. The study focused on the substrates of the coastal areas of the Barents Sea coast (sandy soil in the littoral zone and soil in the coastal zone). The objects of study are the territories on the coast of the Pechenga Bay (near the settlement of Pechenga) and the Kola Bay (the middle part of the bay near the settlements of Belokamenka and Roslyakovo; the southern part of the bay near the settlement of Kola) (Figure 1). The middle part of the Kola Bay is located on the border of the subzones of the forest–tundra (birch crooked forest) and the northern taiga (with a predominance of pine forests), plots of the Pechenga Bay—in the forest–tundra subzone. These are narrow fjord bays typical of the western part of the Barents Sea coast. Despite the similarity, these territories experience anthropogenic pressure of different strength and nature, which, apparently, can lead to serious disruption of local ecosystems. On the coast of the Kola Bay, there is the city of Murmansk, with a population of about 282,000 people, a seaport, industrial enterprises, oil storage facilities, shipyards, and other. The list of the main issuers includes more than 20 enterprises of the Ministries of Defense, Transport, and Housing and Utilities, whose industrial and domestic wastewater flows into the Kola Bay. Significant quantities of pollutants are discharged into the bay—oil products, suspended solids, synthetic surfactants, heavy metals, phenols, nitrogen and phosphorus compounds, etc. Pechenga Bay is experiencing less stress. On its banks are the settlements of Pechenga and Liinakhamari. At the same time, high concentrations of nickel, chromium, lead, copper, organochlorine compounds, and polyaromatic hydrocarbons were noted in the bottom sediments of the Pechenga Bay [12]. For this reason, we are concerned about the state of the natural ecosystems on the Barents Sea coast of the Kola Peninsula and sought to identify excessive anthropogenic interference by assessing a number of characteristics of the soil microbiome.

### 2.3. Sampling Procedure

At each site (object of research), representative sampling points were selected, taking into account vegetation and type of coast. Samples were taken in five replicates. A total of 55 samples were taken and analyzed. For microbiological analysis, soil samples weighing 20–30 g were collected from a depth of 0–10 cm into aseptic plastic containers, which were stored at −18 °C for fluorescence microscopy and at −70 °C for molecular analyzes. Characteristics of the sites are shown in Table 1. 

### 2.4. Biomass of Prokaryotes

The total number of prokaryotes was determined using direct microscopy with a Zeiss Axioskop 2 plus (Oberkochen, Germany) luminescent microscope (×100 lens, oil immersion). Preparations from the soil suspension were stained with acridine orange [13]. Soil (or plant material) samples (1 g) were placed into flasks with 100 mL of sterile water. To desorb cells from the surface of soil particles, the soil suspension was treated with ultrasound using a UDNZ-1 device (2 min, 22 kHz, 0.44 A). Subsequent staining of the preparations with acridine orange was carried out according to the following procedure [14]: 10 μL of the suspension was applied to the glass and distributed over an area of 2 cm × 2 cm; then the glass was fixed in a burner flame and stained with acridine orange (in the dye-to-water ratio of 1:10,000, 2–4 min) immediately before viewing under a microscope with a UV light source. Six preparations were prepared from each sample, in each of which cells in 30 fields of view were counted. The calculation of the number of bacterial cells per 1 g of the substrate was carried out according to the formula

NMA = S_1_ × a × n/v × S_2_ × c × 10^6^,

where N is the number of cells per 1 g of substrate; S_1_ is the area of preparation, μm^2^; a is the number of cells in one field of view (averaged from all preparations); n is the dilution rate of the bacterial mixture, mL; V is the volume of the drop applied to the glass, mL; S_2_ is the area of the field of view, μm^2^; and C is the weighed substrate specimen, 1 g.

### 2.5. Fungal Biomass

The number of fungal propagules and the length of fungal mycelium were determined by luminescence microscopy using a Zeiss Axioskop 2 plus microscope (Oberkochen, Germany) at a magnification of 400. Soil suspension preparations (1:100 dilution) were stained with fluorescent dye calcofluor white [14].

Cell desorption from the soil was performed using a vortex MSV-3500 (Latvia) at 3500 rpm for 10 min. The (10 μL) was applied to the glass and distributed over an area of 2 cm × 2 cm, then the glass was fixed in a burner flame and stained with CW (in the dye-to-water ratio of 1:10,000) for 15–20 min immediately before viewing under a microscope with a UV light source. Three glass preparations were prepared from each soil sample; in each, cells were counted for 90 fields of view. The calculation of the number of fungal cells per 1 g of the substrate was carried out according to the formula

M = ((4an) × p) × 10^10^,

where M is the number of cells per 1 g of soil; a is the average number of cells in a field of view; p is the area of the field of view, μm^2^; and n is the dilution rate, mL. 

The length of the fungal and actinomycetal mycelia in 1 g of soil was calculated as follows:

NMA = S_1_ × a × n/v × S_2_ × c × 10^6^,

where S_1_ is the area of the preparation (μm^2^); a is the average length of mycelium fragments in the field of view (μm); n is the dilution index of the suspension (mL); v is the volume of the drop applied to the glass (ml); v is the volume of the drop applied to the glass (mL); S_2_ is the area of the microscope field of view (μm^2^); c is the sample portion (d). Fungal biomass (mg/g soil) was calculated assuming that the spore density was 0.837 g/cm^3^, and the mycelium density was 0.628 g/cm^3^ [13]. The content of fungal biomass per gram of dry soil was calculated considering its moisture content. Soil and manure DNA and RNA extraction and reverse transcription were carried out according to the method of Semenov and coauthors [15].

Total DNA was extracted and purified from 0.25 g of each spatial soil or manure replicate using the DNeasy PowerSoil Kit (Qiagen, Stockach, Germany). Total RNA was extracted and purified from 2 g of all samples, except soils of sampling 4, using the RNeasy PowerSoil Total RNA Kit (Qiagen, Stockach, Germany) and phenol/chloroform/isoamyl alcohol 25:24:1 saturated with 10 mM Tris (final pH 8.0) and 1 mM EDTA. The homogenization step was performed with a Precellys 24 homogenizer (Bertin Technologies, Bertonneux, France), program 5 (30 s, 6500 rev/min). Co-extracted DNA was removed from RNA samples using RNase-free DNase (Sigma-Aldrich, St. Louis, MO, USA). DNA and RNA quality was estimated by electrophoresis in agarose gels (1% *w*/*v* in TAE) with further visual DNA and RNA detection using the Gel Doc XR + System (Bio-Rad Laboratories, Hercules, CA, USA,). Total RNA was used as the template for cDNA synthesis using the MMLV RT kit (Evrogen Ltd., Moscow, Russia). Extracted DNA, RNA, and cDNA samples were stored at −70 °C until further analyses.

Archaeal, bacterial, and fungal gene quantification by qPCR were carried out according to the method of Semenov and coauthors [15].

For manure and each soil sample, 1 μL of DNA from three replications was used separately to quantify the copy numbers of archaeal, bacterial, and fungal ribosomal genes. Similarly, the copy numbers of bacterial gene transcripts were quantified in cDNA replicates. Gene and gene transcript abundances were estimated using EvaGreen Supermix (Bio-Rad, Hercules, CA, USA). Primers Eub338/Eub518 [16] (Lane 1991), arc915/arc1059 [17] (Yu et al. 2005), and ITS1f/5.8 S [18] (Fierer et al. 2005) were applied for bacteria, archaea, and fungi, respectively. Cloned fragments of *Escherichia coli*, FG-07 *Halobacterium salinarum*, and *Saccharomyces cerevisiae* Meyen 1 B-D1606 ribosomal operons were used to prepare standard solutions of known concentrations. All reactions were performed in a C1000 Thermal Cycler with the CFX96 Real-Time System (Bio-Rad Laboratories, USA, Hercules, California) using the following protocol: 3 min at 95 °C, followed by 49 cycles of 95 °C for 10 s, 50 °C for 10 s, and 72 °C for 20 s. Melting curve analysis was done to check amplicon length. Gene copy numbers were estimated using a regression equation for each assay relating the cycle threshold (Ct) value to the known number of copies in the standard solutions. All sample and standard reactions were performed in triplicate, and average values were calculated.

### 2.6. Statistical Analysis

Statistical data processing was carried out using Microsoft Office Excel 2020 and Statistica 10.0 programs.

## 3. Results

### 3.1. Number of rRNA Gene Copies

**Figure 2:**
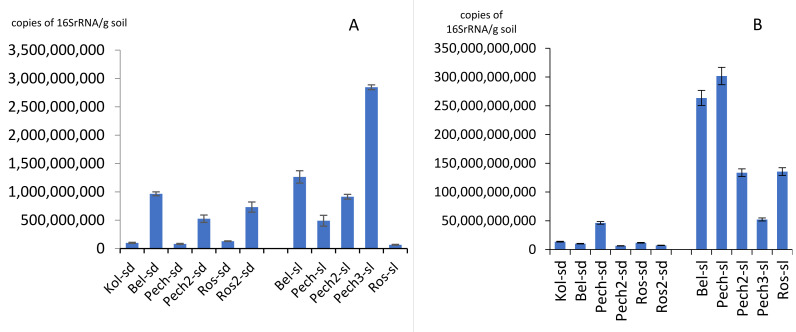
Number of 16S rRNA gene copies of archaea (**A**) and bacteria (**B**).

The number of 16S rRNA gene copies of archaea varied from 6.68 × 10^7^/g soil in the Roslyakovo soil (sample Ros-sl) to 2.85 × 10^9^/g soil in the second beach of Pechenga (sample Pech3-sl) (Figure 2A). For sandy soil samples on the Barents Sea coast, the number of archaeal ribosomal genes varied from 8.17 × 10^7^ to 9.65 × 10^8^/g soil; in the coastal soil—from 6.69 × 10^7^ to 9.65 × 10^9^/g soil (Figure 2A). Values of about 10^7^ gene copies/g soil were also noted in the Pechenga sand (sample Pech-sd). In general, the smallest number of archaea was found on coast in the village Roslyakovo, the largest—in the village Pechenga. For most of the samples (63.4% of all studied samples), the number of archaea was about 10^8^ copies of gene/g soil.

The number of 16S rRNA gene copies of bacteria varied from 6.47 × 10^9^/g soil on the second beach of the Pechenga sandy soil (sample Pech2-sd) to 3.02 × 10^11^/g soil in the soil under the Pechenga grass (sample Pech-sl) (Figure 2B). Values of about 10^9^ gene copies/g soil were also noted in the flooded sand of Roslyakovo (sample Ros2-sd). For most of the samples (9 out of 11 or 81.8% of all studied samples), the number of copies of ribosomal genes of bacteria was on the order of 10^10^–10^11^ copies of genes/g soil. The difference in the number of ribosomal copies of bacterial genes in sandy soil and coastal soil was more pronounced than for the archaea group: in sand it was an order of magnitude lower than in soil. Thus, number of 16S rRNA gene copies of bacteria in sandy soil varied from 6.47 × 10^9^ to 4.63 × 10^10^/g soil; and in the coastal soil, from 5.23 × 10^10^ to 3.02 × 10^11^/g of soil. The smallest number of bacteria, such as archaea, was found on the Roslyakovo coast, the largest number—in Pechenga.

**Figure 3:**
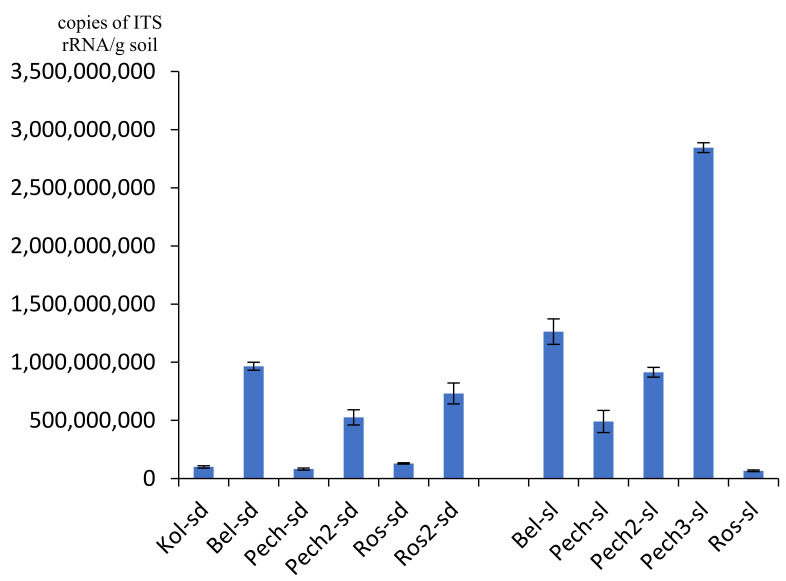
Number of ITS rRNA gene copies of fungi.

The number of ITS rRNA gene copies of fungi varied from 4.28 × 10^7^/g soil in the flooded sand of Roslyakovo (sample Ros-sl) to 4.46 × 10^9^/g soil in the soil under the Pechenga grass (sample Pech-sl3) (Figure 3). Values of about 10^7^ gene copies/g soil were also noted in the Kola sand (sample Kol-sd). For most samples (9 out of 11 or 81.8% of all studied samples), the number of fungi was on the order of 10^8^–10^9^ gene copies/g of soil. The number of ITS rRNA gene copies of fungi, such as bacteria, in sandy soil was an order of magnitude less than in the coastal soil and varied from 4.28 × 10^7^ to 4.96 × 10^8^/g soil, while in soil it varied from 1.07 × 10^9^ to 4.46 × 10^9^/g soil. The largest number of fungi, as well as other groups of microorganisms, was isolated in the substrates of the Pechenga coast, the smallest—in the Roslyakovo area.

### 3.2. Microbial Biomass 

**Figure 4:**
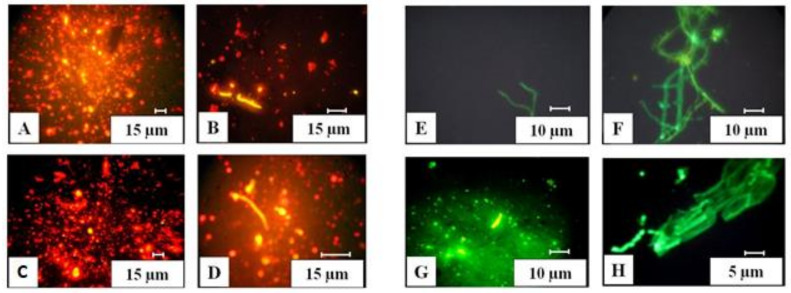
Prokaryotes and fungi under a fluorescent microscope: (**A**,**C**) prokaryotic cells; (**B**,**D**) actinomycete mycelium; (**E**,**F**) mycelium of fungi; (**G**) macrospore of *Fusarium* sp.; (**H**) massive budding of yeast in plant tissue.

The biomass of fungi in studied coastal substrates varied from tens to hundreds of mg/g (Table 2, Figure 4). In sandy soil, it varied from 0.032 to 0.292 mg/g soil; in coastal soil, from 0.021 to 0.715 mg/g soil. In general, the mass of mycobiota in sandy soil at all sampling points was approximately the same, with the exception of Roslyakovo, where it was an order of magnitude greater and amounted to 0.292 mg/g. The maximum mass of mycobiota (0.715 mg/g soil) was recorded in the soil of the coastal territory of Pechenga. The biomass of mycobiota in Kola, Belokamenka, and Roslyakovo was an order of magnitude smaller and comparable with that in sandy soil. For most (64% or 7 out of 11) samples, the biomass of mycobiota was small and varied from 0.021 to 0.073 mg/g soil). However, in the remaining 36% of the samples, fungal biomass was much higher (from 0.292 to 0.715 mg/g soil). Thus, the biomass of Kola and Belokamenka fungi (up to 0.058 mg/g soil) was much less than for Roslyakovo (0.120 mg/g soil) and Pechenga (0.342 mg/g soil).

The proportion of mycelium, an active component of fungal biomass, in the studied samples varied greatly from 25% to 89%. The length of the mycelium of fungi varied from several to hundreds of meters per gram of soil. The minimum of mycelium (up to complete absence) was noted in sandy soil near Pechenga (Pech2-sd), as well as in soil and sand of Roslyakovo (Ros-sl, Ros2-sd). On the Pechenga coast, all samples of the coastal soil showed a high content of fungal mycelium (504.22, 386.55, and 240.02 m/g soil for Pech2-sl, Pech3-sl, Pech-sl, respectively). There was slightly less mycelium in the sandy substrate of Roslyakovo (195.30 m/g soil) and Belokamenka (190.21 m/g soil).

The proportion of thin (less than 3 μm in diameter) mycelium of fungi in the studied soils was relatively high (up to 42%). In most samples, the number of unicellular fungal propagules (spores and yeasts) was 10^4^ cells/g soil; however, in coastal soil of Pechenga (Pech2-sl), their number reached 10^5^ cells/g soil. Most (from 65% to 100%) of mycobiota propagules were represented by specimens of small sizes, 2–3 microns. The smallest proportion of small propagules (65.3–87.8%) was found in the same samples where large spores and yeasts with a characteristic size of 5 μm were found—in sandy soil of Belokamenka (Bel-sd), in soil of Pechenga (Pech2-sl, Pech3-sl), and in the flooded sand of Roslyakovo (Ros2-sd). An abundance of large propagules (in the samples where they were registered) was about 10^3^ cells/g of soil. About 68% of the propagules were round in shape, with a smooth surface: 15%—round and rough; 12%—oval with a smooth surface; 5%—oval with irregularities.

In general, the coastal areas of Kola and Belokamenka turned out to be the poorest locations (0.050 and 0.058 mg/g soil, respectively) in terms of mycobiota biomass. Roslyakovo was much richer in this respect—0.120 mg/g of soil. Most of the fungi were found in Pechenga—0.342 mg/g of soil.

The biomass of prokaryotes in the sandy soil varied from 0.63 to 2.84 µg/g of the substrate and in the soil—from 2.13 to 5.69 µg/g of soil (Table 3, Figure 4). The lowest biomass values were found in sandy soil on the coast of Kola (Kol-sd) and Belokamenka (Bel-sd). The highest biomass of prokaryotes was recorded in the soil of the Pechenga coast (Pech2-sl). On average, the mass of prokaryotes was minimal (0.63 μg/g soil) in Kola and maximum (2.40 μg/g soil) in Pechenga. The biomass of prokaryotes in most samples was represented mainly (from 74.7% to 98.6%) by unicellular forms. However, in the soil (Pech2-sl) and sand (Pech-sd) of Pechenga, the proportion of actinomycete mycelium reached significant values of 57.3% and 66.4%, respectively. The length of the mycelium of actinomycetes ranged from 0.77 to 88.18 m/g soil. No filamentous prokaryotes were found in the Kola sand (Kol-sd). For most of the samples, the hyphae length of actinomycetes did not exceed several m/g of soil. Only in some samples taken on the Pechenga coast (Pech-sl, Pech2-sl, Pech-sd) did the extent of actinomycete mycelium reach tens of m/g of soil.

The number of prokaryotes in the studied soils ranged from hundreds of millions to a billion cells per gram of soil (Table 3). The smallest values (1.87 × 10^8^ cells/g soil) were found in the sand on the Pechenga coast (Pech-sd). The maximum of prokaryotes (1.40 × 10^9^ cells/g soil) was found in the soil on the coast of Pechenga (Pech-sl). Half of the samples were characterized by the number of prokaryotes 10^8^, and half by 10^9^ cells/g soil. Most (up to 61%) of prokaryotic cells were small nanoforms. As well as for mycobiota, Kola and Belokamenka were themselves poor locations (0.63 and 1.53 μg/g soil, respectively) in terms of prokaryotic biomass. Roslyakovo was much richer in this respect—2.09 μg/g of soil. Most prokaryotes were found in Pechenga—2.40 mg/g soil.

## 4. Discussion

Due to active anthropogenic activity on land and sea in the Kola Peninsula region, local ecosystems are severely disturbed. Communities of microorganisms quickly respond to external influences; therefore, their qualitative and quantitative indicators can be indicators of the state of ecosystems. For this reason, the aim of the study was to compare the state of indicators of the subaqueous (marine) and soil (terrestrial) microbiome of the Barents Sea coast of the Kola Peninsula. Previously, the microbe of substrates on the Barents Sea coast of the Kola Peninsula was not investigated by real-time PCR and luminescence microscopy. The originality of the work lies in the simultaneous use of methods of classical and molecular microbiology to test one hypothesis. This study determined the content of copies of ribosomal genes of archaea, bacteria, and fungi, as well as the reserves and structure of the biomass of soil microorganisms (biomass of fungi and prokaryotes, the length and diameter of the mycelium of fungi and actinomycetes, the proportion of mycelium in the biomass, the number of spores and prokaryotic cells, the proportion of small and large fungal propagules, and the morphology of mycobiota spores).

It was shown that the number of copies of the ribosomal 16S rRNA genes of archaea and ITS rRNA of fungi in the analyzed samples varied from 10^7^ to 10^9^ /g soil. This is a fairly high value, given the northern geographic location of the research objects. The number of copies of the 16S rRNA ribosomal genes of bacteria on average was 2 orders of magnitude higher than for archaea and fungi. We assume that the low amount of organic matter in local soils stimulates the growth of bacteria, since this group of microorganisms is capable of carrying out many stages of biogeochemical cycles of nutrients. The biomass of fungi varied from tens to hundreds of mg/g of soil, and the biomass of prokaryotes, from tenths to units of μg/g of soil. Such a significant difference is probably associated with the cell size of these groups of microorganisms. Fungal cells are much larger than those of archaea and bacteria, while their physical density is approximately the same. This is why the mass of fungi was greater than the mass of prokaryotes. The length of the mycelium of fungi varied from units to hundreds of meters per gram of soil, and the length of the mycelium of actinomycetes—from tenths to tens of meters per gram of soil. This pattern is easily explained by the fact that usually the body of fungi consists almost entirely of mycelium, while the organism of many bacteria is unicellular. Thus, bacteria prevailed in abundance in the studied substrates of the Kola Peninsula, and fungi prevailed in weight.

The number of copies of the 16S rRNA ribosomal genes of archaea in coastal soils is, on average, 2–3 orders of magnitude lower than in forest and urban soils of the northern taiga zone of the Kola Peninsula and is comparable with that in the forest–tundra zone [19]. We believe that the coastal areas of the Kola Peninsula are less favorable for archaea than the urban environment in the subarctic climate due to the “heat island” effect [20,21] and large reserves of organic matter [22,23] in the urban soils. It should be noted that, despite the lack of nutrients in sandy soil, the number of copies of archaean genes does not differ significantly from that in the soil, in contrast with other groups of microorganisms—bacteria and fungi.

The number of 16S rRNA gene copies of bacteria in the studied coastal soils is lower than in the urban and forest soil of northern taiga and forest–tundra zones of Kola Peninsula [19]. Probably due to the vulnerability of bacteria to pollutants in the urban environment [24], the abundance of these prokaryotes noticeably increases in background landscapes, especially in cold climates [24,25]. In the sandy soil, the number of bacterial gene copies is 1–2 orders of magnitude lower than in the coastal soil.

The number of copies of the ribosomal genes ITS rRNA of fungi in the studied samples is, on average, an order of magnitude lower than in the urban soils of the northern taiga zone of the Kola Peninsula, but is comparable with the soils of the forest–tundra zone [26]. Considering that the main limiting factor in the development of fungi is the availability of available organic matter, we can assume that the number of fungi is higher in urban soils due to the greater variety of organic substrates.

Thus, bacteria dominated in the microbial complex of soils and grounds on the Barents Sea coast of the Kola Peninsula. For most samples (82%), their number was about 10^10^–10^11^ copies of genes/g of soil, while in 82% of samples the number of fungi was about 10^8^–10^9^ copies of genes/g of soil, while in 63.4% of samples, the number of archaea was about 10^8^ copies of genes/g soil. The largest number of ribosomal copies of genes of all groups of microorganisms was noted on the coast of the settlement Pechenga, the smallest—in Roslyakovo. Apparently, this result was obtained because the Pechenga Bay and the soils of its environs have the least anthropogenic load, limiting the growth of microorganisms.

For some objects, real-time PCR and luminescence microscopy methods obtained opposite data, such as biomass was either low or the number of copies of ribosomal genes was too high, or vice versa. This can be explained by an irregular distribution of genetic material over cells of microorganisms [26,27]. Thus, for example, in some cells the amount of DNA may be low, while in other cells of the same size it is large. Such heterogeneity is usually associated with the characteristics of the life cycle of the microorganism.

Fungal biomass. In the studied substrates of the Barents Sea coast, biomass of mycobiota was less than in background landscapes of the Kola Peninsula [5,7], but it was 1.5–2 times higher than in soils of the city of Apatity, which is located farther south in the Murmansk region [21]. Apparently, this may be due to such limiting factors as heavy metal pollution by powerful enterprises in the cities of the Kola Peninsula [28,29]. The length of fungal mycelium in the studied samples was comparable to that for the soils at the Kandalaksha aluminum plant (also located on the Kola Peninsula) [19], but greater compared with these creatures’ length in soils of the Rybachiy Peninsula (northern part of the Kola Peninsula) [30] and in the north of Novaya Zemlya archipelago [31]. We assume that such a difference in the length of the fungal mycelium can primarily be explained by the severity of the climatic conditions. The proportion of thin (less than 3 μm in diameter) mycelium in the studied soils was relatively high (up to 42%) compared with the percentage of that in urban soils of Apatity and Murmansk [21]. Perhaps this may be due to the “heat island” effect characteristic of cities beyond the Arctic Circle [20,21]. Extra warmth from a city can stimulate the growth of soil fungi. Basidiomycete buckle mycelium of fungi was found rarely (about 5% of all hyphae), and only in surface organogenic layers, which was 4–8 times lower compared with the proportion of such mycelium in urban soils of the Kola Peninsula [21]. The fact may indirectly indicate a low number of mycorrhizal symbioses [32].

Despite the fact that soil is the natural habitat of most of the fungi, it often creates unfavorable conditions for the development of mycobiota propagules [33]. Therefore, it is necessary to estimate not only the proportion of active biomass—mycelium—but also the percentage of resting cells [34]. The number of unicellular fungal propagules in the order of values corresponded to their number in the soils of some cities of the Kola Peninsula [21] and to soils of the Kandalaksha aluminum plant [19], as well as to the soils of the Rybachiy peninsula [30], but higher compared with values for soils of Novaya Zemlya [31]. Thus, we can conclude that the Kola Peninsula is a more hospitable place for fungi than the relatively more northerly Novaya Zemlya archipelago. The abundance of unicellular mycobiota propagules, on whole, correlated with the biomass and length of mycelium of the mycobiota; however, this pattern was not observed for the flooded sandy substrate of Roslyakovo (Ros-sd) (Table 1). In this sample, budding was noted in some of cells, which may indicate a significant activity of oligotrophic yeast [34]. Yeasts are mainly single-celled, so it is difficult to distinguish them from dormant fungal propagules (spores, conidia, etc.) using the method of fluorescence microscopy [35]. The absence of large propagules with a diameter of 5–7 μm for most samples can be associated with extreme climatic conditions [33,36] typical for north of the Kola Peninsula [37].

Kola and Belokamenka sites were themselves poor, while Roslyakovo and Pechenga sites were the richest locations in fungi. We believe that this may be due to the high degree of pollution of the soils of these locations with oil products, which some fungi can use as a nutrient substrate (Table 1).

Biomass of prokaryotes. The abundance and biomass of prokaryotes in the studied soils of the Barents Sea coast of the Kola Peninsula was low, as in other Arctic territories—Taimyr [38], Novaya Zemlya [31,36], and Franz Josef Land [39]. This result may indicate an equally high ability of prokaryotes to survive in polar regions, regardless of their geographic location. However, the maximum of prokaryotes (about 10^9^ cells/g of soil) in the analyzed soils is comparable in an order of magnitude with that for soddy-podzolic soils of central Russia [40,41,42]. This fact indicates a high potential for the biological activity of microorganisms in some loci, even for the Arctic territories. Average biomass for the studied locations differed significantly (up to 3.8 times) (Table 2), which may be due to the abundance of organic carbon in soil, as noted in other works [43,44]. The length of actinomycete mycelium in most of the studied samples was small (a few meters per gram of soil), although the proportion of a representative of this biological order of Gram-positive bacteria was relatively large for most polar ecosystems [45,46]. Therefore, we believe that actinobacteria in the studied samples are predominantly represented by unicellular forms. The length of the mycelium of actinomycetes for the analyzed soils was somewhat lower compared with the urban soils of the northern taiga and forest tundra zones of the Kola Peninsula [21]. Presumably, this may be due to a smaller variety of organic substrates and a low level of heat in the background natural ecosystems as compared with anthropogenic ones. Most (up to 61%) of prokaryotic cells were represented by small nanoforms, which is typical for polar ecosystems [47]. Usually, smaller microorganisms are better adapted to various extreme factors.

The number of prokaryotes, as well as fungi, was minimal in the sites of Kola and Belokamenka, but it was maximal in the sites of Roslyakovo and Pechenga. This may be due to the high content of oil products in the soils. Depending on the physical and chemical properties, oil products can both inhibit the growth of microorganisms and serve as a nutrient substrate for them.

In general, the abundance of petroleum products did not significantly affect the number of all groups of microorganisms studied. We believe that this is primarily due to the non-critical concentration of hydrocarbons for the vital activity of most soil archaea, bacteria, and fungi. The main factor influencing the growth and development of microorganisms was the content of organic carbon in the soil, which was consistent with the work of many microbiologists [48,49,50].

## 5. Conclusions

For the first time, using a complex of classical and molecular genetic microbiological methods, the soil microbiome of the Barents Sea coast of the Kola Peninsula was characterized. It was shown that, despite the extreme position on the mainland land of Fennoscandia, local soils have a significant number of microorganisms, on which the productivity of ecosystems largely depends. For the analyzed sandy soils, the number of copies of ribosomal genes was maximal, but the biomass of microorganisms was minimal, while the coastal soils were characterized by an inverse relationship. The largest number of copies of ribosomal genes in the substrates of the coastal area was found for bacteria. The number of the ribosomal gene copies of fungi and archaea was 2 orders of magnitude less than that of bacteria. Mycobiota prevailed in the microbial biomass and ranged from 90% to 97%. The proportion of fungal mycelium varied from 25% to 89%. Most of the propagules of mycobiota were represented by specimens of small sizes, 2–3 microns. Thus, even in the oligotrophic conditions of the Subarctic zone, microorganisms are able to actively develop, colonizing substrates that are not very suitable for other forms of life. We believe that such microbiological indicators as the number of ribosomal genes, the number of cells, the length of the mycelium, and the biomass reserves can be effectively used to detect anthropogenic disturbances in polar ecosystems.

## Figures and Tables

**Table 1:** Characteristics of soils on the Barents Sea coast of the Kola Peninsula.

Locality	Coordinates	Sample Code	TOC,%	pH	Petroleum Hydrocarbon Content, mg/kg	Description of the Site
Kola	68°53′07.3″ N 33°02′22.6″ E	Kol-sd	0.81	6.81	711	The foot of the coastal ledge; rocky beach with deposits of sand between stones
Belokamenka	69°04′35.7″ N 33°10′12.8″ E	Bel-sl	13.25	5.57	568	Soil: 0–7 cm—organogenic, deeper than 7 cm—sand; *Leymus arenarius, Lathyrus aleuticus, Mertensia maritima, Festuca arenaria, Ligusticum scoticum, Achillea apiculata, Atriplex glabriuscula, Chamaenerion angustifolium*
		Bel-sd	0.24	6.43	211	Sandy and gravelly beach with a lot of stones
Pechenga	69°34′38.6″ N 31°13′58.5″ E	Pech-sl	22.26	5.94	130	Soil: 0–3 cm—organogenic, deeper than 3 cm—sand; *Rumex confertus, Caltha palustris, Chamaenerion angustifolium, Festuca arenaria*
		Pech-sd	1.25	5.42	59	Sandy and gravelly beach
	69°34′09.0″ N 31°13′50.4″ E	Pech2-sl	4.74	5.11	97	Soil: 0–2 cm—organogenic, deeper than 2 cm—medium loam (hydrogen sulfide decay is in the loam); *Carex* sp.
		Pech2-sd	0.10	7.19	46	Sandy beach
		Pech3-sl	9.74	5.70	174	Soil: 0–5 cm—organogenic, deeper than 5 cm—sand; *Solidago lapponica, Chamaenerion angustifolium, Salix *sp.*, Festuca arenaria*
Roslyakovo	69°03′22.4″ N 33°13′39.3″ E	Ros-sl	17.44	6.86	203	Soil: 0–5 cm—organogenic, deeper than 5 cm—sand and gravel; *Achillea apiculate*, *Chamaenerion angustifolium, Salix *sp.*, Taraxacum officinale, Festuca *sp.**
		Ros-sd	1.32	7.22	252	The foot of the coastal ledge; rocky beach with deposits of sand and gravel between stones
		Ros2-sd	1.25	7.20	245	The foot of the coastal ledge; rocky beach with deposits of gravel between stones

Note: -sd, sand samples; -sl, soil samples.

**Table 2:** The structure of the fungal biomass.

Sample Description		Mycelium (Mainly d = 3 μm)			Spores (Diameter, μm)						Total Spore Biomass, mg/g Soil	The Portion of Small (2–3 μm) Spores by Weight,%	Total Biomass of Fungi, mg/g Soil	Average Biomass of Fungi by Location, mg/g Soil
		Biomass, mg/g	Length, m	Proportion of Mycelium in Total Biomass,%	2		3		5					
					Number, Cells/g × 10^4^	Biomass, mg/g	Number, Cells/g × 10^4^	Biomass, mg/g	Number, Cells /g × 10^3^	Biomass, mg/g				
Kola	Kol-sd	0.027 ± 0.004	21.59 ± 2.96	54.0	4.32 ± 0.59	0.015 ± 0.002	0.65 ± 0.09	0.008 ± 0.001	-	-	0.023 ± 0.004	100	0.050 ± 0.009	0.050 ± 0.009
Belokamenka	Bel-sl	0.024 ± 0.003	190.21 ± 26.08	32.9	3.88 ± 0.53	0.013 ± 0.002	1.62 ± 0.22	0.019 ± 0.003	1.74 ± 0.34	0.017 ± 0.002	0.049 ± 0.007	65.3	0.073 ± 0.011	0.058 ± 0.010
	Bel-sd	0.015 ± 0.002	11.54 ± 1.58	34.9	2.59 ± 0.35	0.009 ± 0.001	1.62 ± 0.22	0.019 ± 0.003	-	-	0.028 ± 0.004	100	0.043 ± 0.006	
Pechenga	Pech-sl	0.303 ± 0.045	240.02 ± 32.91	86.3	6.04 ± 0.83	0.020 ± 0.003	2.43 ± 0.34	0.028 ± 0.004	-	-	0.048 ± 0.007	100	0.351 ± 0.053	0.342 ± 0.051
	Pech2-sl	0.637 ± 0.094	504.22 ± 69.13	89.1	9.06 ± 1.25	0.031 ± 0.004	3.24 ± 0.45	0.037 ± 0.005	1.04 ± 0.20	0.010 ± 0.001	0.078 ± 0.012	87.2	0.715 ± 0.107	
	Pech-sd	0.018 ± 0.002	14.51 ± 1.99	43.9	3.45 ± 0.47	0.012 ± 0.002	0.97 ± 0.13	0.011 ± 0.001	-	-	0.023 ± 0.004	100	0.041 ± 0.006	
	Pech2-sd	0.008 ± 0.001	6.33 ± 0.87	25.0	2.59 ± 0.36	0.009 ± 0.001	1.30 ± 0.17	0.015 ± 0.002	-	-	0.024 ± 0.004	100	0.032 ± 0.004	
	Pech3-sl	0.488 ± 0.072	386.55 ± 53.00	85.6	4.74 ± 0.65	0.016 ± 0.002	4.86 ± 0.65	0.056 ± 0.008	1.04 ± 0.20	0.010 ± 0.001	0.082 ± 0.012	87.8	0.570 ± 0.086	
Roslyakovo	Ros-sl	-	-	0.0	3.02 ± 0.42	0.010 ± 0.001	0.97 ± 0.13	0.011 ± 0.001	-	-	0.021 ± 0.003	100	0.021 ± 0.003	0.120 ± 0.018
	Ros-sd	0.250 ± 0.037	195.30 ± 26.78	85.6	4.74 ± 0.65	0.016 ± 0.002	2.27 ± 0.30	0.026 ± 0.004	-	-	0.042 ± 0.006	100	0.292 ± 0.044	
	Ros2-sd	-	-	0.0	3.02 ± 0.42	0.010 ± 0.001	1.94 ± 0.26	0.023 ± 0.003	1.39 ± 0.27	0.013 ± 0.002	0.046 ± 0.007	71.7	0.046 ± 0.007	

Note: “-“ sign means “not found”.

**Table 3:** Structure of the prokaryote’s biomass.

Description Sample		Number of Prokaryotic Cells, × 10^8^ Cells/g	Biomass of Unicellular Prokaryotes, μg/g Soil	Actinomycete Mycelium Length, m/g	Biomass of Actinomycete Mycelium, μg/g	The Share of Mycelium in the Total Biomass, %	Total Biomass of Prokaryotes, μg/g Soil	Average Biomass of Prokaryotes by Location, μg/g Soil
Kola	Kol-sd	2.96 ± 0.43	0.63 ± 0.10	-	-	0.0	0.63 ± 0.10	0.63 ± 0.10
Belokamenka	Bel-sl	10.00 ± 1.47	2.12 ± 0.32	8.22 ± 1.24	0.30 ± 0.05	12.4	2.42 ± 0.38	1.53 ± 0.24
	Bel-sd	2.48 ± 0.36	0.53 ± 0.08	2.84 ± 0.43	0.10 ± 0.01	15.9	0.63 ± 0.10	
Pechenga	Pech-sl	13.99 ± 2.05	2.97 ± 0.44	25.24 ± 3.82	0.92 ± 0.16	23.8	3.90 ± 0.62	2.40 ± 0.38
	Pech2-sl	11.49 ± 1.68	2.43 ± 0.36	88.18 ± 13.33	3.22 ± 0.54	57.3	5.69 ± 0.90	
	Pech-sd	1.87 ± 0.27	0.40 ± 0.06	22.12 ± 3.34	0.81 ± 0.14	66.4	1.22 ± 0.19	
	Pech2-sd	3.80 ± 0.56	0.81 ± 0.12	4.17 ± 0.63	0.15 ± 0.03	15.6	0.96 ± 0.15	
	Pech3-sl	9.99 ± 1.47	2.10 ± 0.31	0.77 ± 0.12	0.03	1.4	2.13 ± 0.34	
Roslakovo	Ros-sl	10.40 ± 1.53	2.18 ± 0.32	3.04 ± 0.46	0.11 ± 0.01	4.8	2.29 ± 0.36	2.09 ± 0.33
	Ros-sd	10.05 ± 1.48	2.11 ± 0.31	1.97 ± 0.30	0.72 ± 0.14	25.4	2.84 ± 0.45	
	Ros2-sd	5.27 ± 0.78	1.06 ± 0.16	2.67 ± 0.40	0.09 ± 0.01	7.8	1.15 ± 0.18	

Note: “-“ sign means “not found”.

## Data Availability

Not applicable.

## References

[1] Rühland K.M., Paterson A.M., Keller W., Michelutti N., Smol J.P. (2013). Global warming triggers the loss of a key Arctic refugium. Proc. R. Soc. B Biol. Sci..

[2] Pereverzev V.N. (2011). Soils and soil cover of the Kola Peninsula: History and current state of research. Bull. Kola Sci. Cent. Russ. Acad. Sci..

[3] Evdokimova G.A., Mozgova N.P. (2001). Comparative characteristics of microbial biomass of AI-FE-humus podzols of the Kola Peninsula. Eurasian Soil Sci..

[4] Pereverzev V.N. (2007). Forest soils of the Kola Peninsula. For. Green Build. West. Sib..

[5] Korneykova M.V. (2018). Comparative analysis of the number and structure of the complexes of microscopic fungi in tundra and taiga soils in the north of the Kola Peninsula. Eurasian Soil Sci..

[6] Nikitin D.A., Semenov M.V., Chernov T.I., Ksenofontova N.A., Zhelezova A.D., Ivanova E.A., Kozlov D.N., Khitrov N.B., Stepanov A.L. (2021). Microbiological indicators of ecological functions of soils (review). Eurasian Soil Sci..

[7] Fierer N., Wood S.A., de Mesquita C.P. (2021). How microbes can, and cannot, be used to assess soil health. Soil Biol. Biochem..

[8] Marshall G.J., Vignols R.M., Rees W.G. (2016). Climate change in the Kola Peninsula, Arctic Russia, during the last 50 years from meteorological observations. J. Clim..

[9] Hättestrand C., Clark C.D. (2006). The glacial geomorphology of Kola Peninsula and adjacent areas in the Murmansk Region, Russia. J. Maps.

[10] Jacoby R., Peukert M., Succurro A., Koprivova A., Kopriva S. (2017). The role of soil microorganisms in plant mineral nutrition—current knowledge and future directions. Front. Plant Sci..

[11] Koroleva N.E., Chinenko S.V., Sortland E.B. (2011). Communities of marches, beaches and coastal floodplain ephemeretum of the Murmansk, Tersk and Vostok of the Kandalaksha coast (Murmansk region). Phyto-Divers. East. Eur..

[12] Gorin S.L., Leman V.N. (2017). Hydrological regime and pollution in the basin and in the water area of the Pechenga Bay (Varanger Fjord of the Barents Sea) according to long-term observations of the hydrometeorological service. Tr. VNIRO.

[13] Polyanskaya L.M., Zvyagintsev D.G. (2005). The content and composition of microbial biomass as an index of the ecological status of soil. Eurasian Soil Sci..

[14] Zvyagintsev D.G. (1991). Methods of Soil Microbiology and Biochemistry.

[15] Semenov M.V., Krasnov G.S., Semenov V.M., Ksenofontova N., Zinyakova N.B., van Bruggen A.H. (2021). Does fresh farmyard manure introduce surviving microbes into soil or activate soil-borne microbiota?. J. Environ. Manag..

[16] Stackebrandt E., Goodfellow M. (1991). Nucleic Acid Techniques in Bacterial Systematics.

[17] Yu Y., Lee C., Kim J., Hwang S. (2005). Group-specific primer and probe sets to detect methanogenic communities using quantitative real-time polymerase chain reaction. Biotechnol. Bioeng..

[18] Fierer N., Jackson J.A., Vilgalys R., Jacksson R.B. (2005). Assessment of soil microbial community structure by use of taxon-specific quantitative PCR assays. Appl. Environ. Microbiol..

[19] Korneikova M.V., Nikitin D.A. (2021). Qualitative and quantitative characteristics of the soil microbiome in the zone of influence of emissions from the Kandalaksha aluminum plant. Eurasian Soil Sci..

[20] McLean M.A., Angilletta M.J., Williams K.S. (2005). If you can’t stand the heat, stay out of the city: Thermal reaction norms of chitinolytic fungi in an urban heat island. J. Therm. Biol..

[21] Korneikova M.V., Nikitin D.A., Dolgikh A.V., Soshina A.S. (2020). Soil mycobiota of the city of Apatity (Murmansk region). Mikol. Fitopatol..

[22] Vodyanitsky Y.N. (2015). Organic matter in urban soils (literature review). Eurasian Soil Sci..

[23] Vasenev V., Kuzyakov Y. (2018). Urban soils as hot spots of anthropogenic carbon accumulation: Review of stocks, mechanisms and driving factors. Land Degrad. Dev..

[24] Lysak L.V., Lapygina E.V. (2018). The diversity of bacterial communities in urban soils. Eurasian Soil Sci..

[25] Hui N., Jumpponen A., Francini G., Kotze D.J., Liu X., Romantschuk M., Strömmer R., Setälä H. (2017). Soil microbial communities are shaped by vegetation type and park age in cities under cold climate. Environ. Microbiol..

[26] Shoemaker W.R., Lennon J.T. (2018). Evolution with a seed bank: The population genetic consequences of microbial dormancy. Evol. Appl..

[27] Zhelezova A., Chernov T., Tkhakakhova A., Xenofontova N., Semenov M., Kutovaya O. (2019). Prokaryotic community shifts during soil formation on sands in the tundra zone. PLoS ONE.

[28] Demyanenko D.A. Analysis of the state of the environment in the Murmansk region. Proceedings of the Current Problems and Prospects for the Development of State Statistics in Modern Conditions.

[29] Evdokimova G.A., Mozgova N.P., Korneikova M.V. (2014). Content and toxicity of heavy metals in soils of zone affected by gas-air emissions from the Pechenganikel plant. Eurasian Soil Sci..

[30] Korneikova M.V., Redkina V.V., Myazin V.A., Fokina N.V., Shalygina R.R. (2019). Microorganisms of soils of the Rybachiy Peninsula. Proc. Kola Sci. Cent. Russ. Acad. Sci. USA.

[31] Nikitin D.A., Lysak L.V., Kholod S.S., Mergelov N.S., Dolgikh A.V., Goryachkin S.V. (2021). Biological activity of soils on Northern (Novaya Zemlya). Eurasian Soil Sci..

[32] Fomicheva O.A., Polyanskaya L.M., Nikonov V.V., Lukina N.V., Orlova M.A., Isaeva L.G., Zvyagintsev D.G. (2006). Population and biomass of soil microorganisms in old-growth primary spruce forests in the Northern Taiga. Eurasian Soil Sci..

[33] Garrett S.D. (2016). Soil Fungi and Soil Fertility: An Introduction to Soil Mycology.

[34] Nikitin D.A., Marfenina O.E., Maksimova I.A. (2017). Using the succession approach in studying the species composition of microscopic fungi and the content of fungal biomass in Antarctic soils. Mikol. Fitopatol..

[35] Rozali S.N., Milani E.A., Deed R.C., Silva F.V. (2017). Bacteria, mold and yeast spore inactivation studies by scanning electron microscope observations. Int. J. food Microbiol..

[36] Nikitin D.A., Lysak L.V., Mergelov N.S., Dolgikh A.V., Zazovskaya E.P., Goryachkin S.V. (2020). Microbial Biomass, Carbon Stocks, and CO_2_ Emission in Soils of Franz Josef Land: High-Arctic Tundra or Polar Deserts?. Eurasian Soil Sci..

[37] Popova O.N., Shcherbina Y.F. (2012). Climatogeophysical characteristics of the Kola Polar region. Ekol. Cheloveka.

[38] Schmidt N., Bölter M. (2002). Fungal and bacterial biomass in tundra soils along an arctic transect from Taimyr Peninsula, central Siberia. Polar Biol..

[39] Nikitin D.A., Semenov M.V., Semikolennykh A.A., Kachalkin A.V., Ivanova A.E. (2019). Biomass of fungi and species diversity of the cultivated mycobiota of soils and substrates in Northbrook Island (Franz Josef Land). Mikol. Fitopatol..

[40] Khabibullina F.M., Kuznetsova E.G., Vaseneva I.Z. (2014). Micromycetes in podzolic and bog-podzolic soils in the middle taiga subzone of northeastern European Russia. Eurasian Soil Sci..

[41] Nikitin D.A., Chernov T.V., Zhelezova A.D., Tkhakakhova A.K., Nikitina S.A., Semenov M.V., Xenofontova N.A., Kutovaya O.V. (2019). Seasonal Dynamics of Microbial Biomass in Soddy-Podzolic Soil. Eurasian Soil Sci..

[42] Polyanskaya L.M., Sukhanova N.I., Chakmazyan K.V., Zvyagintsev D.G. (2012). Changes in the structure of soil microbial biomass under fallow. Eurasian Soil Sci..

[43] Bakermans C., Emili L.A. (2019). Terrestrial systems of the Arctic as a model for growth and survival at low temperatures. Model Ecosystems in Extreme Environments.

[44] Pastor A., Freixa A., Skovsholt L.J., Wu N., Romaní A.M., Riis T. (2019). Microbial Organic Matter Utilization in High-Arctic Streams: Key Enzymatic Controls. Microb. Ecol..

[45] Dubrova M.S., Lubsanova D.A., Makarova E.P., Kozhevin P.A., Manucharova N.A., Zenova G.M. (2011). Psychrotolerant actinomycetes in soils of the tundra and northern taiga. Mosc. Univ. Soil Sci. Bull..

[46] Millán-Aguiñaga N., Soldatou S., Brozio S., Munnoch J.T., Howe J., Hoskisson P.A., Duncan K.R. (2019). Awakening ancient polar Actinobacteria: Diversity, evolution and specialized metabolite potential. Microbiology.

[47] Kudinova A.G., Lysak L.V., Soina V.S., Mergelov N.S., Dolgikh A.V., Shorkunov I.G. (2015). Bacterial communities in the soils of cryptogamic barrens of East Antarctica (the Larsemann Hills and Thala Hills oases). Eurasian Soil Sci..

[48] Ivashchenko K., Ananyeva N., Vasenev V., Sushko S., Seleznyova A., Kudeyarov V. (2019). Microbial C-availability and organic matter decomposition in urban soils of megapolis depend on functional zoning. Soil Environ..

[49] Grosse G., Harden J., Turetsky M., McGuire A.D., Camill P., Tarnocai C., Frolking S., Schuur E.A.G., Jorgenson T., Marchenko S. (2011). Vulnerability of high-latitude soil organic carbon in North America to disturbance. J. Geophys Res. Biogeosci..

[50] Lorenz K., Lal R. (2015). Managing soil carbon stocks to enhance the resilience of urban ecosystems. Carbon Manag..

